# Voice in Parkinson's Disease: A Machine Learning Study

**DOI:** 10.3389/fneur.2022.831428

**Published:** 2022-02-15

**Authors:** Antonio Suppa, Giovanni Costantini, Francesco Asci, Pietro Di Leo, Mohammad Sami Al-Wardat, Giulia Di Lazzaro, Simona Scalise, Antonio Pisani, Giovanni Saggio

**Affiliations:** ^1^Department of Human Neurosciences, Sapienza University of Rome, Rome, Italy; ^2^IRCCS Neuromed Institute, Pozzilli, Italy; ^3^Department of Electronic Engineering, University of Rome Tor Vergata, Rome, Italy; ^4^Department of Allied Medical Sciences, Aqaba University of Technology, Aqaba, Jordan; ^5^Neurology Unit, Fondazione Policlinico Universitario Agostino Gemelli IRCCS, Rome, Italy; ^6^Department of System Medicine UOSD Parkinson, University of Rome Tor Vergata, Rome, Italy; ^7^Department of Brain and Behavioral Sciences, University of Pavia, Pavia, Italy; ^8^IRCCS Mondino Foundation, Pavia, Italy

**Keywords:** Parkinson's disease, hypokinetic dysarthria, voice analysis, machine learning, L-Dopa

## Abstract

**Introduction:**

Parkinson's disease (PD) is characterized by specific voice disorders collectively termed hypokinetic dysarthria. We here investigated voice changes by using machine learning algorithms, in a large cohort of patients with PD in different stages of the disease, OFF and ON therapy.

**Methods:**

We investigated 115 patients affected by PD (mean age: 68.2 ± 9.2 years) and 108 age-matched healthy subjects (mean age: 60.2 ± 11.0 years). The PD cohort included 57 *early-stage* patients (Hoehn &Yahr ≤ 2) who never took L-Dopa for their disease at the time of the study, and 58 *mid-advanced-stage* patients (Hoehn &Yahr >2) who were *chronically-treated* with L-Dopa. We clinically evaluated voices using specific subitems of the Unified Parkinson's Disease Rating Scale and the Voice Handicap Index. Voice samples recorded through a high-definition audio recorder underwent machine learning analysis based on the support vector machine classifier. We also calculated the receiver operating characteristic curves to examine the diagnostic accuracy of the analysis and assessed possible clinical-instrumental correlations.

**Results:**

Voice is abnormal in *early-stage* PD and as the disease progresses, voice increasingly degradres as demonstrated by high accuracy in the discrimination between healthy subjects and PD patients in the *early-stage* and *mid-advanced-stage*. Also, L-dopa therapy improves but not restore voice in PD as shown by high accuracy in the comparison between patients OFF and ON therapy. Finally, for the first time we achieved significant clinical-instrumental correlations by using a new score (LR value) calculated by machine learning.

**Conclusion:**

Voice is abnormal in *early-stage* PD, progressively degrades in *mid-advanced-stage* and can be improved but not restored by L-Dopa. Lastly, machine learning allows tracking disease severity and quantifying the symptomatic effect of L-Dopa on voice parameters with previously unreported high accuracy, thus representing a potential new biomarker of PD.

## Introduction

Patients with Parkinson's disease (PD) often complain of a variable impairment of voice emission including hypophonia, mono-pitch and mono-loudness speech, hypokinetic articulation, collectively called hypokinetic dysarthria (1–4). Parkinsonian patients may manifest voice disorders in the early stage of the disease, with growing evidence showing voice impairment occurring even in the prodromal phase of PD (2, 5–9). Also, voice typically worsens over the course of the disease leading to severe voice impairment in more advanced stages of PD (1, 2). Furthermore, the standardized clinical assessment of voice in PD is currently based only on qualitative evaluation (i.e., a specific subitem of the Unified Parkinson's Disease Rating Scale—UPDRS) (2, 10) thus precluding the objective assessment of the voice impairment in this disorder.

Over recent years, quantitative approaches based on spectral analysis have been developed to examine objectively voice samples (11). Spectral analysis in patients with PD allowed to demonstrate several abnormalities in specific voice features such as reduced fundamental frequency and harmonics-to-noise ratio, and increased jitter and shimmer (3, 12–16). The human voice however, represents a complex phenomenon characterized by high-dimensional data based on an exponential number of features. Accordingly, besides the independent examination through spectral analysis of specific voice features (i.e., fundamental frequency), more advanced techniques able to analyse and dynamically combine and high-dimensional datasets of voice features such as machine-learning algorithms (17–23) would improve significantly the accuracy of the objective classification of voice samples in PD. Indeed, machine learning has allowed to classify voice impairment objectively and automatically in a number of neurologic disorders, with previously unreported high accuracy (19, 21, 22).

To date, concerning the application of machine learning analysis in PD, only a few preliminary studies in rather small and clinically heterogeneous cohorts of patients have been reported (24–26). It is therefore important to examine instrumentally voice impairment in a large and clinically well-characterized cohort of PD. Also, it is relevant to verify whether machine learning can recognize the effect of disease severity by discriminating patients in different stages of the disease. Still, given that the symptomatic effect of L-Dopa on voice is still largely a matter of debate (1, 10, 27–33), it is relevant to compare the instrumental voice analysis with machine learning in patients under and not under L-Dopa treatment.

We here investigated voice in a large and clinically well-characterized cohort of patients with PD. Then, to examine the effect of disease severity on voice, we compared voices collected in patients in *early* and *mid-advanced* stage of PD. Still, to investigate the effect of L-Dopa on voice, we compared patients OFF and ON therapy. To verify the effect of the specific speech tasks, we compared voice recordings during the emission of a vowel and a sentence, according to standardized procedures (19, 21, 22). We assessed the sensitivity, specificity, positive and negative predictive values, and accuracy of all diagnostic tests and calculated the area under the receiver operating characteristic (ROC) curves. Lastly, by providing a machine learning measure of voice impairment severity for each patient, we also assessed possible clinical-instrumental correlations. Our hypothesis is that machine learning analysis of speech samples is able to discriminate PD patients from controls, patients in *early* and *mid-advanced stages*, and finally patients OFF and ON therapy, with previously unreported high accuracy.

## Methods

### Subjects

We enrolled a total of 115 patients affected by PD (68.2 ± 9.2 years, range 47–91 years) and 108 age-matched healthy subjects (HS) (60.2 ± 11.0 years). Participants were recruited at the IRCCS Neuromed Institute and at the Department of Systems Medicine, Tor Vergata University of Rome, Italy. All participants (HS and PD patients) were native Italian speakers and non-smokers. None of the participants reported bilateral/unilateral hearing loss, respiratory disorders, other non-neurologic disorders affecting the vocal cords. Participants gave written informed consent, which was approved by the institutional ethics committee (0026508/2019), according to the Declaration of Helsinki.

The clinical diagnosis of PD was made according to current standardized clinical criteria (34). Symptoms and signs associated with PD were scored using Hoehn & Yahr scale (H&Y), UPDRS part III (10). None of the patients manifested atypical parkinsonian symptoms. In all participants (HS and PD patients), we assessed cognitive function and mood using the Mini-Mental State Evaluation (MMSE) (35), the Hamilton Depression Rating Scale (HAM-D) (36) and the Frontal Assessment Battery (FAB). None of the patients were treated with deep brain stimulation or infusional therapies. The clinical evaluation of speech was achieved by two independent raters using two separate clinical scales: (1) the Voice Handicap Index (VHI), Italian version (37), which consists of a patient-based, self-assessed, 30-item scale examining the functional, physical, and emotional aspects of voice disorders; (2) the specific item for speech evaluation included in the UPDRS-III scale (UPDRS-III-v) (10).

The study cohort was designed to include a subgroup of 57 *early stage* patients with PD (H&Y scores ≤ 2) (38) who never took L-Dopa for their disease at the time of the study *(drug naïve)*(64.2 ± 8.6 years), and a subgroup of 58 *mid-advanced-stage* patients (H&Y scores >2) (38) who were *chronically-treated* with L-Dopa (72.1 ± 8.1 years). We evaluated 31 out of 58 *mid-advanced-stage* patients (71.4 ± 8.7 years) when OFF (after at least 12 h of L-Dopa withdrawal) and ON therapy (1–2 h after the intake of L-Dopa). Participant demographic and clinical features are reported in Table 1.

**Table 1:** Demographic and clinical features of HS and PD.

	**Age (years)**	**Weight (kg)**	**Height> (cm)**	**DD (years)**	**MMSE**	**HAM-D**	**FAB**	**H&Y**	**UPDRS-III OFF**	**UPDRS-III ON**	**UPDRS-III-v OFF**	**UPDRS-III-v ON**	**VHI OFF**	**VHI ON**
PD (whole group)	68.2 ± 9.2	71.8 ± 11.6	172.1 ± 9.4	5.6 ± 4.7	28.4 ± 2.1	3.5 ± 1.8	16.5 ± 1.4	2.2 ± 0.8	22.3 ± 14.2	–	1.8 ± 1.1	–	16.7 ± 16.9	–
*Early-stage* PD	64.2 ± 8.6	71.8 ± 10.6	172.9 ± 9.8	2.1 ± 0.9	28.9 ± 1.1	3.2 ± 2.0	16.6 ± 1.0	1.5 ± 0.4	12.1 ± 4.1	–	0.9 ± 0.7	–	7.3 ± 4.9	–
*Mid-advanced-stage* PD	72.1 ± 8.1	71.9 ± 12.6	171.2 ± 9.0	9.0 ± 4.4	28.0 ± 2.6	3.9 ± 1.6	16.4 ± 1.6	2.8 ± 0.4	32.3 ± 13.5	28.3 ± 13.8	2.7 ± 0.6	2.4 ± 0.5	25.9 ± 19.2	20.0 ± 17.7
HS	70.3 ± 10.3	68.5 ± 10.6	169.0 ± 10.1	–	29.0 ± 0.8	3.3 ± 1.7	16.6 ± 1.1	–	–	–	–	–	–	–

*DD, disease duration; MMSE, Mini-Mental State Evaluation; HAM-D, Hamilton Depression Rating Scale; FAB, Frontal Assessment Battery; H&Y, Hoehn and Yahr Scale for assessment stage of PD; HS, healthy subjects; PD, patients with Parkinson's disease; UPDRS-III, Unified Parkinson's Disease Rating Scale part III; UPDRS-III-v, Unified Parkinson's Disease Rating Scale part III, voice impairment subitem; VHI, Voice Handicap Index; OFF, not-under the effect of L-Dopa; ON, under the effect of L-Dopa. Results are expressed as average ± standard deviation*.

### Voice Recordings

Voice recordings were performed by asking participants to produce a specific speech task with their usual voice intensity, pitch, and quality. The speech tasks consisted of the sustained emission of a close-mid front unrounded vowel /e/ for at least 5 s and of the emission of a standardized Italian sentence (19, 22). Voice recordings were collected by using a high-definition audio-recorder H4n Zoom (Zoom Corporation, Tokyo, Japan), connected with a Shure WH20 Dynamic Headset Microphone (Shure Incorporated, USA), which was placed at a distance of 5 cm from the mouth. Voice samples were recorded in linear PCM format (.wav) at a sampling rate of 44.1 kHz, with 16-bit sample size.

### Machine Learning Analysis

Each voice sample underwent feature extraction pre-process by using OpenSMILE (audEERING GmbH, Germany) (39). For each voice sample, we extracted 6,139 voice features included in the INTERSPEECH2016 Computational Paralinguistics Challenge (IS ComParE 2016) feature dataset (39). To identify a subset of the most relevant features, the extracted voice features underwent feature selection pre-process using the Correlation Features Selection algorithm (CSF) (40). CFS was applied in order to select (uncorrelated) voice features highly correlated with the class. As a result, redundant and/or irrelevant features were removed from the original dataset. All the selected features were then ranked in order of relevance, by measuring the information gain concerning the class, through the Information Gain Attribute Evaluation (IGAE) algorithm, which is based on the Pearson's correlation method. To further increase the accuracy of results, we used the discretization pre-process, which is an optimization procedure consisting in calculating the best splitting point from the two classes and assigning a binary value to the features. Discretization was achieved using the Fayyad & Irani's discretization method, according to standardized procedures.

Given the relatively small dataset analyzed in the study, the Support Vector Machine (SVM) classifier based on linear kernel was used to achieve a binary classification, reducing the likelihood for “overfitting.” We used only the first 30 most relevant features ranked by the IGAE (22). This approach was applied to reduce the number of selected features needed to perform the machine learning analysis, in according to standardized procedures (18, 19, 21, 22). A list of the first 30 features which represent functionals applied to audio low-level descriptors (LLDs)—extracted from the vowel and the sentence for the comparison between HS and PD is reported in Table 2. The SVM was trained using the sequential minimal optimization method. Both the procedures of feature selection and classification were performed through MATLAB (MathWorks, USA). The training was performed using an optimization procedure aimed to find the best hyperparameter values for binary classification (i.e., box constraint “C” value, for linear kernel). Different combinations of hyperparameter values were tested by using an optimization scheme that seeks to minimize the model classification error (41, 42).

**Table 2:** List of the first 30 selected features for the comparison between HS and PD.

**Vowel**				**Sentence**		
**Ranking position**	**Families of LLDs**	**LLDs**	**Functionals**	**Families of LLDs**	**LLDs**	**Functionals**
1	RASTA coefficients	Coefficient of band 22	Standard deviation of falling slope	Spectral LLD	Spectral Roll Off point 0.90	Absolute peak range
2	Voicing Related LLD	Fundamental Frequency (fo)	Minimum segment length	Spectral LLD	Spectral Roll Off point 0.50	Inter-quartile 1–3
3	Energy Related LLD	Sum of auditory spectrum	Flatness	Spectral LLD	Spectral Roll Off point 0.50	Quartile 3
4	Spectral LLD	Spectral Flux	Quadratic regression coefficient 1	Energy Related LLD	Zero Crossing Rate	99% percentile
5	RASTA coefficients	Coefficient of band 2	Linear prediction coefficient 4	Spectral LLD	Spectral Variance	Range
6	RASTA coefficients	Coefficient of band 21 (de)	Standard deviation of rising slope	Spectral LLD	Spectral Roll Off point 0.25	Quartile 3
7	Spectral LLD	Spectral Slope (de)	Position of max	Spectral LLD	Spectral Roll Off point 0.25	Linear prediction coefficient 0
8	RASTA coefficients	Coefficient of band 25	Flatness	Spectral LLD	Psychoacoustic Sharpness	1% percentile
9	Spectral LLD	Spectral energy 250–650 Hz	Relative min range	RASTA coefficients	Coefficient of band 8 (de)	Flatness
10	Energy Related LLD	RMS Energy (de)	Linear prediction coefficient 0	Spectral LLD	Spectral Centroid	99% percentile
11	Spectral LLD	Spectral Flux	Standard deviation of falling slope	Spectral LLD	Spectral Roll Off point 0.75	Absolute peak range
12	Voicing Related LLD	Fundamental Frequency (fo)	1% percentile	RASTA coefficients	Coefficient of band 1	Mean of rising slope
13	MFCC	8th Mel Coefficient	Inter-quartile 1–2	Spectral LLD	Spectral Roll Off point 0.25	Quadratic regression coefficient 2
14	RASTA coefficients	Coefficient of band 25 (de)	Gain of linear prediction	MFCC	2nd Mel Coefficient	Quadratic regression quadratic
15	Spectral LLD	Spectral Flux	Range	Spectral LLD	Spectral Roll Off point 0.25	Inter-quartile 2–3
16	Spectral LLD	Spectral Flux	Quadratic regression coefficient 2	Spectral LLD	Spectral Entropy	Range
17	Spectral LLD	Spectral Slope	Gain of linear prediction	Energy Related LLD	Zero Crossing Rate	Standard deviation of rising slope
18	Spectral LLD	Spectral Slope	Standard deviation of rising slope	Spectral LLD	Spectral Roll Off point 0.50	Quadratic regression coefficient 3
19	Spectral LLD	Spectral Variance (de)	Relative peak mean	Voicing Related LLD	Fundamental frequency	Inter-quartile 2–3
20	MFCC	5th Mel Coefficient (de)	Skewness	Spectral LLD	Spectral Entropy	Absolute peak mean
21	RASTA coefficients	Coefficient of band 4 (de)	Skewness	MFCC	3rd Mel Coefficient	1% percentile
22	Energy Related LLD	RMS Energy	Mean of falling slope	Spectral LLD	Spectral Variance	Inter-quartile 2–3
23	Spectral LLD	Spectral Roll Off point 0.75	Linear prediction coefficient 3	RASTA coefficients	Coefficient of band 18	Position of min
24	RASTA coefficients	Coefficient of band 5	Linear prediction coefficient 4	MFCC	3rd Mel Coefficient	Relative peak mean
25	Energy Related LLD	Zero Crossing Rate	Linear prediction coefficient 0	Spectral LLD	Spectral Kurtosis	Absolute peak range
26	MFCC	4th Mel Coefficient (de)	Relative peak range	RASTA coefficients	Coefficient of band 9 (de)	Flatness
27	Voicing Related LLD	Shimmer (Local)	Position of max	RASTA coefficients	Coefficient of band 4	Position of min
28	RASTA coefficients	Coefficient of band 2	Linear prediction coefficient 3	Spectral LLD	Spectral Centroid	1% percentile
29	RASTA coefficients	Coefficient of band 1 (de)	Standard deviation	Spectral LLD	Spectral Skewness	Mean segment length
30	Voicing Related LLD	Shimmer (Local) (de)	Quadratic regression coefficient 2	RASTA coefficients	Coefficient of band 22	Position of min

*The table refers to selected voice features for the comparison between healthy subjects and patients with Parkinson's disease. Ranking of the first 30 features (functionals applied to low-level descriptors—LLDs) extracted using a dedicated software (OpenSMILE) and selected using Information Gain Attribute Evaluation (IGAE) algorithm for the comparison between healthy subjects and the whole group of patients with PD, during the sustained emission of the vowel and sentence. MFCC, mel-frequency cepstral coefficient; de, first derivative of the LLD*.

**Figure 1:**
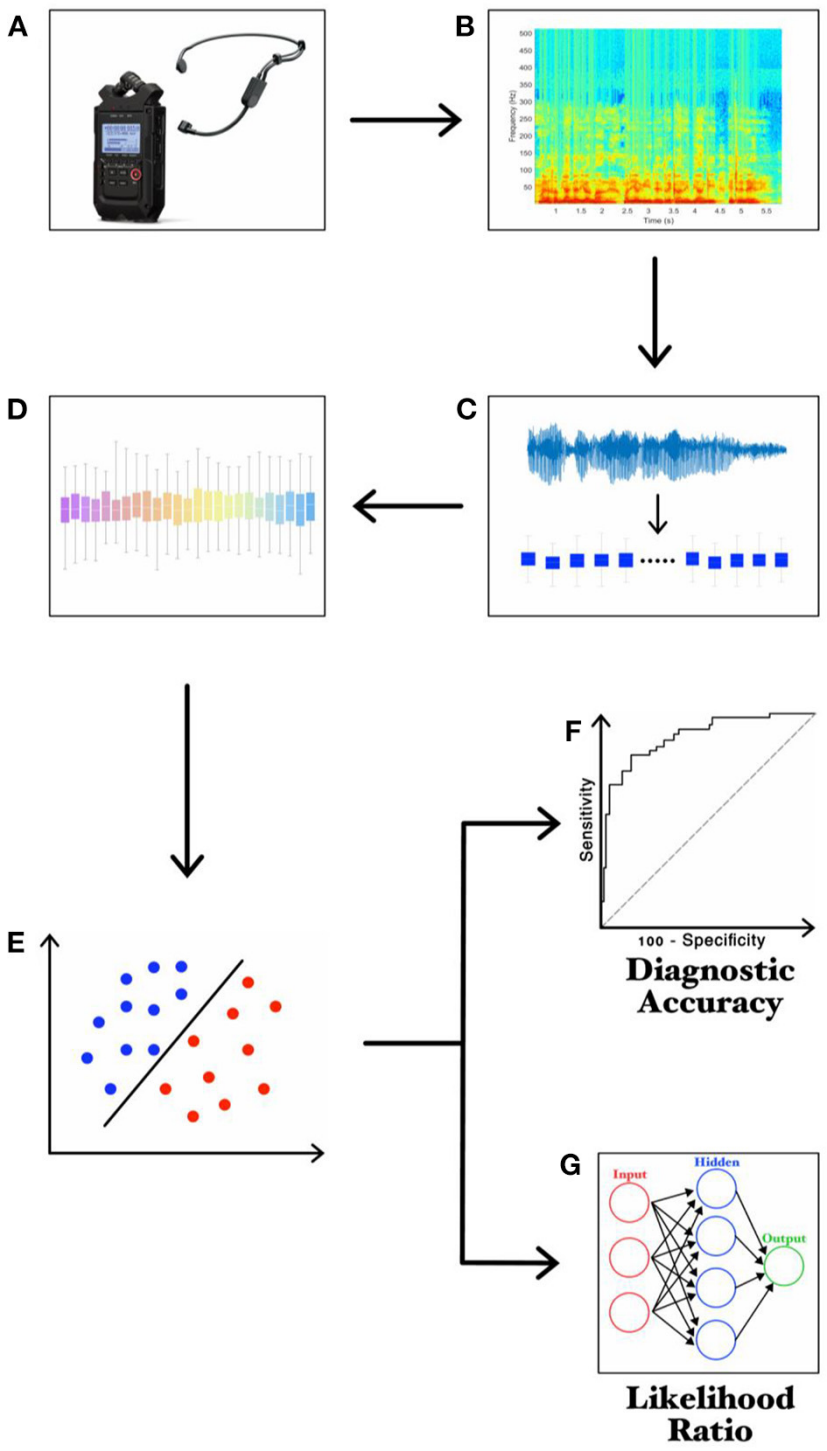
Experimental design. **(A)** recording of voice samples through a high-definition audio recorder; **(B)** narrow-band spectrogram of the acoustic voice signal; **(C)** feature extraction; **(D)** feature selection; **(E)** feature classification; **(F)** ROC curve analysis; **(G)** LR values calculated through ANN.

We performed a further machine learning analysis for clinical-instrumental correlation purposes, after achieving feature extraction and selection, in parallel to the SVM classification procedures. We used a feed-forward artificial neural network (ANN), consisting of a 30-neurons input layer, a 10-neurons hidden layer and a one-neuron output layer. Input for ANN consisted of the first 30 most relevant selected features, which thus matched the 30-neurons input layer. Then, the ANN was trained to calculate a continuous numerical value (the likelihood ratio—LR), ranging from 0 to 1 and reflecting the degree of voice impairment in each patient with PD (i.e., the closer the LRs to 1, the higher the degree of voice impairment). ANN was trained by using the same selected features used to train the SVM. The experimental paradigm is also summarized in Figure 1 (39–42).

### Statistical Analysis

The normality of all parameters was assessed using the Kolmogorov-Smirnov test. The Mann-Whitney *U* test was used to compare demographic and anthropometric parameters in HS and PD patients. The Mann-Whitney U test was also used to compare demographic parameters and clinical scores in *early-stage* and *mid-advanced-stage* patients. The Wilcoxon signed-rank test was used to compare UPDRS-III, UPDRS-III-v, and VHI scores in *mid-advanced-stage* patients when OFF and ON therapy. The Wilcoxon signed-rank test was also used to compare the possible L-Dopa-induced improvement of voice (UPDRS-III-v-ON/OFF^*^100) and motor symptoms (UPDRS-III-ON/OFF^*^100) in *mid-advanced-stage* patients.

ROC analyses were calculated to identify the optimal diagnostic cut-off values to discriminate between HS and PD, *early-stage* and *mid-advanced-stage* patients, and finally *mid-advanced-stage* patients OFF and ON therapy. We reported in detail the Sensibility (Se), Specificity (Sp), Positive Predictive Value (PPV), Negative Predictive Value (NPV), Accuracy (Acc.). Also, we showed the output of the ROC analysis by calculating the Youden Index (YI) and its optimal criterion value, the associated criterion (Ass. Crit.). We also compared the independent ROC curves referring to the emission of the vowel and the sentence.

Spearman's rank correlation coefficient was used to assess correlations between clinical scores and LR values.

A *p*-value < 0.05 was considered statistically significant.

## Results

Demographic and anthropometric parameters were normally distributed in HS, in PD as well as in *early-stage* and *mid-advanced-stage* patients (*p* > 0.05). Weight, height, and BMI were comparable among groups (*p* > 0.05). Mean age was comparable between HS and *mid-advanced-stage* patients (*p* > 0.05), whereas it was higher in HS and *mid-advanced-stage* patients than in *early-stage* patients (*p* < 0.05). MMSE, HAM-D and FAB were comparable among groups (*p* > 0.05 for all comparisons). *Mid-advanced-stage* patients showed higher scores on the H&Y, UPDRS-III, UPDRS-III-v and VHI scales than *early-stage* patients (*p* < 0.05 for all comparisons). The L-Dopa-induced improvement of voice was lower than that in the remaining motor symptoms (*p* < 0.05) (Table 1).

### Voice Impairment in PD

We found that 84% of the patients included in our cohort (97 out of 115 patients) manifested a variable degree of clinically overt voice impairment (UPDRS-III-v ≥1). Also, we found a clinically overt voice impairment in 68% of *early-stage* patients and 100% of *mid-advanced-stage* patients.

**Figure 2:**
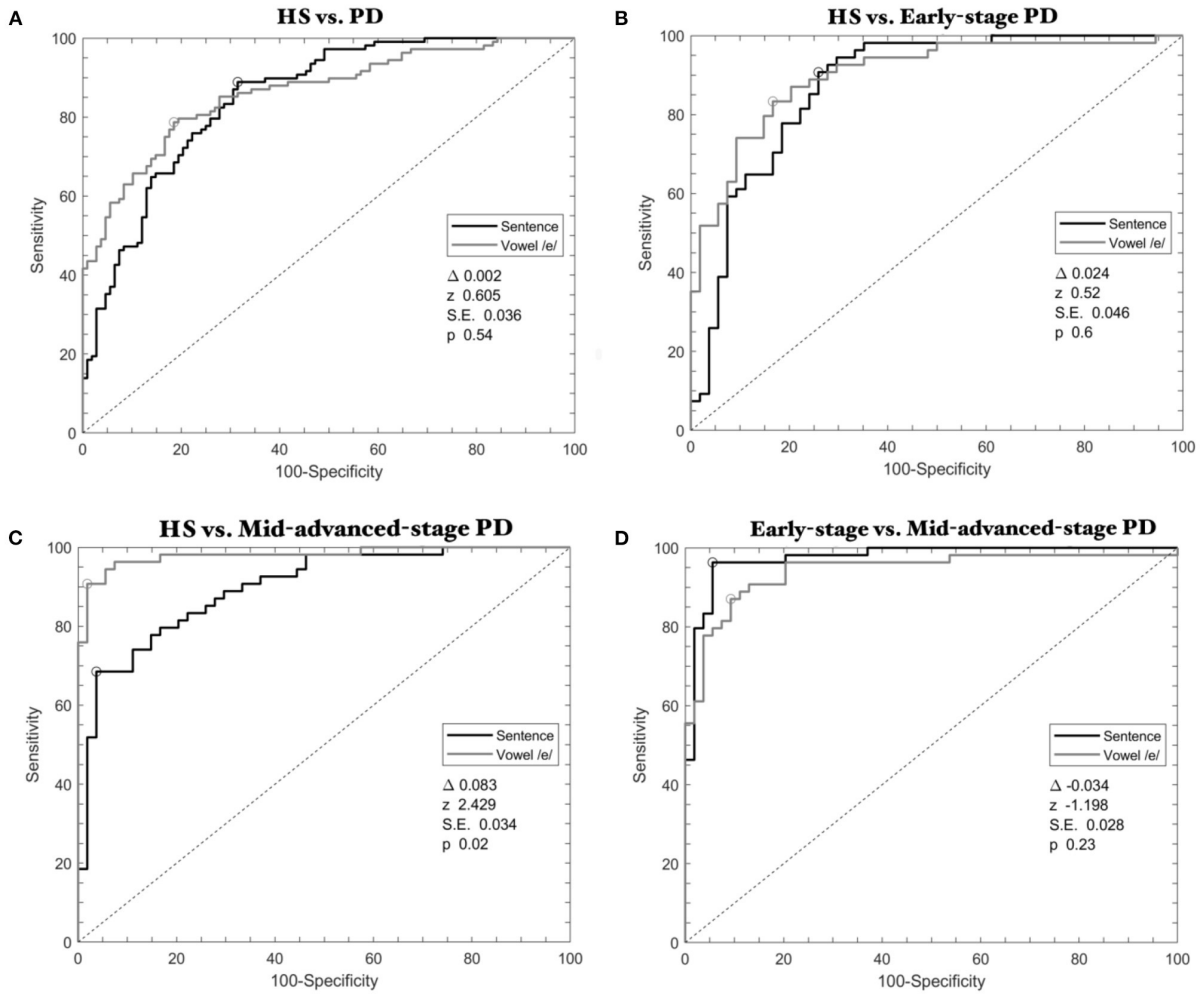
ROC curves calculated through SVM classifier in Parkinson's disease. **(A)** HS vs. the whole group of PD patients; **(B)** HS vs. *early-stage* patients; **(C)** HS vs. *mid-advanced-stage* patients OFF therapy; **(D)** *Early-stage* vs. *mid-advanced-stage* patients OFF therapy. Gray lines refer to the emission of the vowel, whereas black lines refer to the sentence.

Voice samples collected in 7 patients with PD (3 patients from the *early-stage* subgroup and 4 patients from the *mid-advanced-stage* subgroup including voice recordings collected in 2 patients ON and OFF therapy) were excluded from the instrumental analysis owing to file corruption. We first compared voice samples recorded during the emission of vowel and sentence in HS and the whole group of patients. This analysis showed a significant and comparable diagnostic performance between speech tasks (delta-AUC = 0.002, *z* = 0.605, SE = 0.036, *p* = 0.54) (Figure 2A, Table 3).

**Table 3:** Performance of the machine learning algorithm.

**Comparisons**	**Speech-task**	**Instances**	**Cross validation**	**Associated criterion**	**Youden index**	**Se (%)**	**Sp (%)**	**PPV (%)**	**NPV (%)**	**Acc (%)**	**AUC**
HS vs. PD	Vowel	98	10 folds	−0.03	0.60	82.7	77.1	75.0	84.3	79.6	0.870
	Sentence	94	10 folds	0.02	0.57	72.5	84.7	88.0	66.7	77.3	0.848
HS vs. *early-stage* PD	Vowel	67	10 folds	−0.36	0.64	87.0	77.4	74.1	88.9	81.5	0.900
	Sentence	93	10 folds	0.16	0.66	75.8	90.5	92.6	70.4	81.5	0.876
HS vs. mid-a*dvanced-stage* PD	Vowel	100	10 folds	0.16	0.87	92.7	94.3	94.4	92.6	93.5	0.980
	Sentence	82	10 folds	0.18	0.63	82.7	80.4	79.6	83.3	81.5	0.897
*Early-stage* vs. *mid-advanced-stage* PD	Vowel	119	10 folds	0.16	0.76	87.2	88.7	88.9	87.0	88.0	0.934
	Sentence	102	10 folds	0.10	0.85	91.1	94.1	94.4	90.7	92.6	0.981
*Mid-advanced-stage* PD OFF vs. ON	Vowel	22	10 folds	0.02	0.46	69.7	76.0	79.3	65.5	72.4	0.754
	Sentence	6	10 folds	0.03	0.49	71.9	76.9	79.3	69.0	74.1	0.786
HS vs. *mid-advanced-stage* PD ON	Vowel	82	10 folds	0.97	0.66	85.2	80.6	79.3	86.2	82.8	0.913
	Sentence	69	10 folds	−0.01	0.93	96.6	96.6	96.6	96.6	96.6	0.985
*Early-stage* PD vs. *mid-advanced-stage* PD ON	Vowel	71	10 folds	−0.18	0.94	100	93.5	93.1	100	96.6	0.992
	Sentence	78	10 folds	0.62	0.97	100	96.7	96.6	100	98.3	0.999

*Performance of SVM linear classifier elaborating the 30 most relevant selected features during the sustained emission of the vowel and the sentence for seven independent conditions: (1) HS vs. the whole group of PD patients; (2) HS vs. early-stage patients; (3) HS vs. mid-advanced-stage patients; (4) Early-stage vs. mid-advanced-stage patients; (5) Mid-advanced-stage patients OFF vs. ON therapy; (6) HS vs. mid-advanced-stage patients ON therapy; (7) Early-stage patients vs. mid-advanced-stage patients ON therapy. Selected features refer to the number of features able to obtain the best results; instances refer to the number of subjects considered in each comparison; cross validation refers to standardized validation procedures (see methods for details). Se, sensitivity; Sp, specificity; PPV, positive predictive value; NPV, negative predictive value; Acc, accuracy; AUC, area under the curve*.

When discriminating HS and *early-stage* patients, ROC analyses identified high accuracy with comparable results between speech tasks (delta-AUC = 0.024, *z* =0.520, SE = 0.046, *p* = 0.60) (Figure 2B, Table 3).

When comparing HS and *mid-advanced-stage* patients OFF therapy, ROC analyses again showed high classification accuracy but the analysis showed higher results for the vowel than the sentence (delta-AUC = 0.083, *z* = 2.429, SE = 0.034, *p* = 0.02) (Figure 2C, Table 3).

Also, when discriminating *early-stage* and *mid-advanced-stage* patients, ROC curves showed high and comparable results between speech tasks (delta-AUCs = −0.034, *z* = −1.198, SE = 0.028, *p* = 0.23) (Figure 2D, Table 3).

### The Effect of L-Dopa on Voice

We found that pharmacological treatment with L-Dopa induced a significant clinical improvement of both motor and voice impairment, as demonstrated by reduced UPDRS-III (PD-ON: 28.3 ± 13.8; PD-OFF: 32.3 ± 13.5; *z* = −4.9; W = 0; *p* < 0.01), UPDRS-III-v (PD-ON: 2.4 ± 0.5; PD-OFF: 2.7 ± 0.6; *z* = −2.9; W = 0; *p* < 0.05) and VHI scores (PD-ON: 20.0 ± 17.7; PD-OFF: 25.9 ± 21.4; *z* = −4.9; W = 0; *p* < 0.01).

**Figure 3:**
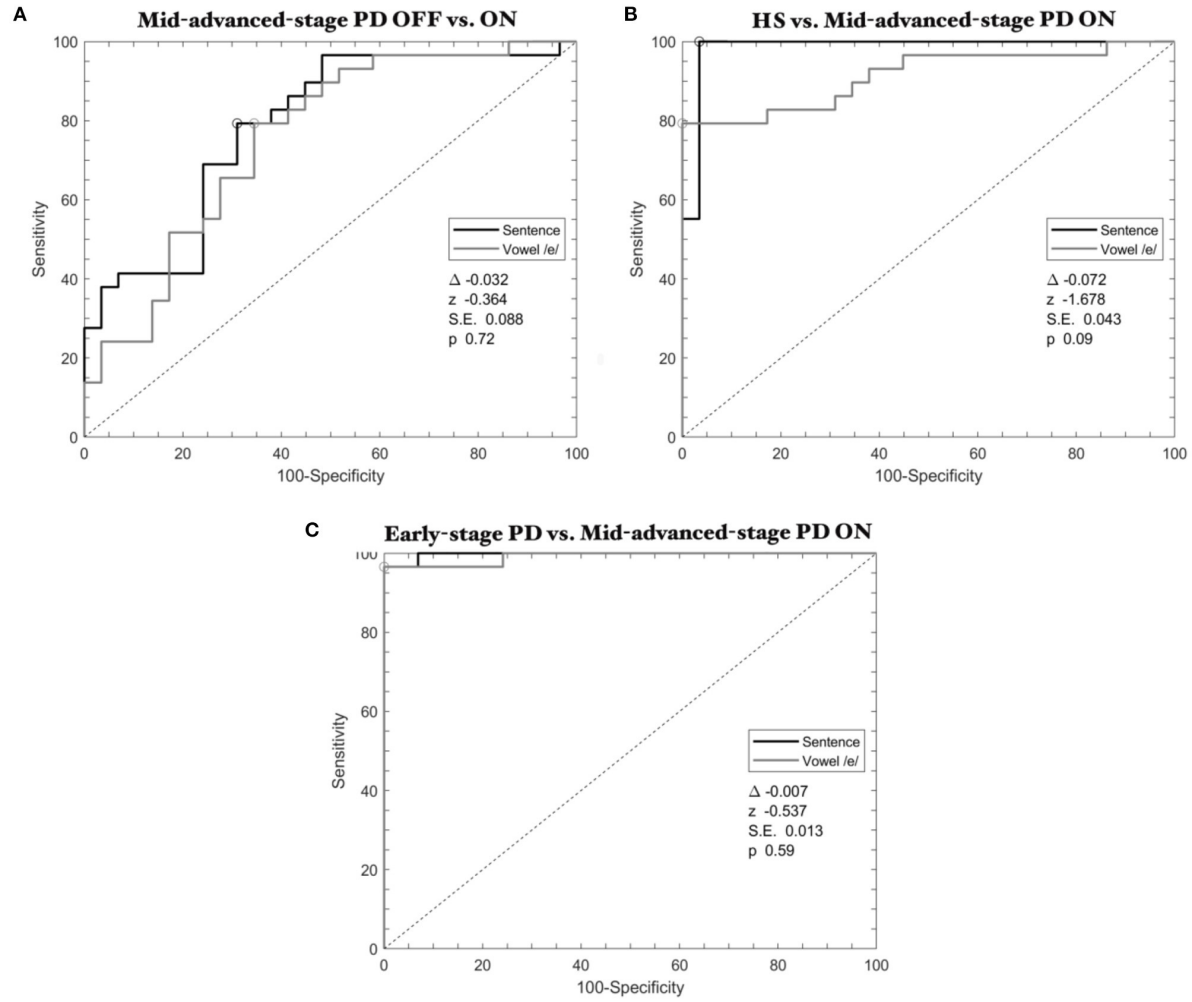
ROC curves calculated through SVM classifier in Parkinson's disease: the effect of L-Dopa. **(A)** *Mid-advanced-stage* patients OFF vs. ON therapy; **(B)** HS vs. *mid-advanced-stage* patients ON therapy; **(C)** *Early-stage* patients vs. *mid-advanced-stage* patients ON therapy. Gray lines refer to the emission of the vowel, whereas black lines refer to the sentence.

When comparing *mid-advanced-stage* patients OFF and ON, ROC analysis showed comparable results between speech tasks with high accuracy (delta-AUC = −0.032, *z* = −0.364, SE = 0.088, *p* = 0.72) (Figure 3A, Table 3).

When discriminating HS and *mid-advanced-stage* patients ON therapy, ROC analysis showed high classification performance (delta-AUC = −0.072, *z* = −1.678, SE = 0.043, *p* = 0.09) (Figure 3B, Table 3).

Finally, concerning the comparison between *early-stage* and *mid-advanced-stage* patients when ON therapy, ROC analysis showed high statistical results for both the speech tasks (delta-AUC = −0.007, *z* = −0.537, SE = 0.013, *p* = 0.59) (Figure 3C, Table 3).

### Correlation Analysis

**Figure 4:**
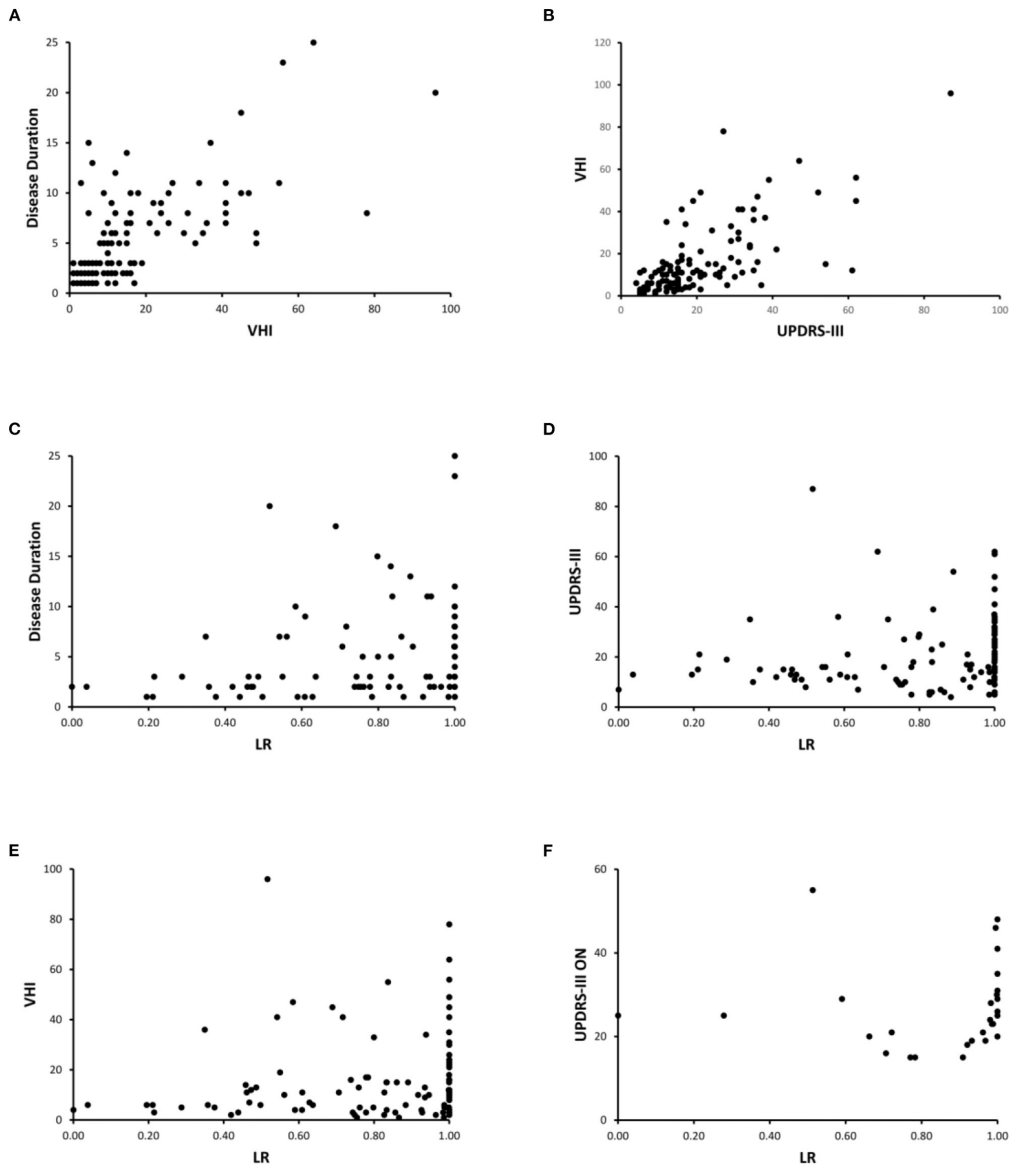
Clinical-instrumental correlations. **(A)** Disease Duration and VHI; **(B)** UPDRS-III and VHI; **(C)** Disease Duration and LRs; **(D)** UPDRS-III and LRs; **(E)** VHI and LRs; **(F)** UPDRS-III ON and LRs. Note that the correlation analysis only refers to the emission of the vowel. Similar results have been achieved when analyzing the emission of a sentence (data not shown). In addition, correlation analysis shown in **(A–E)** refers to the whole group of PD patients, whereas **(F)** shows the correlation assessed in the subgroup of *mid-advanced stage* patients ON therapy.

In the whole group of PD patients, the Spearman test disclosed a positive correlation between disease duration and VHI (*r* = 0.64, *p* < 0.01) (Figure 4A), H&Y and UPDRS-III-v scores (*r* = 0.76, *p* < 0.01), and between H&Y and VHI (*r* = 0.64, *p* < 0.01), i.e., the greater disease duration and disability, the higher impairment of voice. We also found a positive correlation between UPDRS-III and UPDRS-III-v scores (*r* = 0.81, *p* < 0.01), and between UPDRS-III and VHI (*r* = 0.64, *p* < 0.01) (Figure 4B), i.e., the greater disease severity, the higher impairment of voice. Furthermore, there was a positive correlation also between LEDDs and VHI scores (*r* = 0.34, *p* < 0.01), and UPDRS-III-v scores (*r* = 0.44, *p* < 0.01), i.e., the higher LEDDs, the higher impairment of voice. Lastly, MMSE and FAB negatively correlated with VHI scores (*r* = −0.37, *p* < 0.01 and *r* = −0.28, *p* < 0.01, respectively), i.e., the greater cognitive impairment, the higher impairment of voice.

Concerning the clinical-instrumental correlations, we found a positive correlation between LRs collected in the overall group of PD patients and disease duration (*r* = 0.35, *p* < 0.01) (Figure 4C), H&Y (*r* = 0.34, *p* < 0.01), UPDRS-III (*r* = 0.41, *p* < 0.01) (Figure 4D), UPDRS-III-v (*r* = 0.33, *p* < 0.01), and VHI (*r* = 0.33, *p* < 0.01) (Figure 4E). When considering *mid-advanced-stage* PD patients ON therapy, we found a positive correlation between LRs and UPDRS-III scores (*r* = 0.47, *p* < 0.05) (Figure 4F). Accordingly, the higher LR values attributed by machine learning, the higher disease duration, disability, and severity of motor as well as voice symptoms.

## Discussion

We here report the objective and automatic recognition, by means of machine learning, of voice abnormalities in a large and clinically well-characterized cohort of patients with PD. We demonstrated the effect of disease severity on voice changes in PD by discriminating *early-stage* and *mid-advanced-stage* patients. Also, we clarified the effect of L-Dopa on voice in PD by recognizing voice changes in patients OFF and ON therapy. The significant clinical-instrumental correlations further support the high diagnostic accuracy of our voice analysis.

All the subjects here enrolled were non-smokers and native Italian speakers. HS and PD had comparable demographic, anthropometric and cognitive characteristics including MMSE scores corrected for years of education. We recruited a balanced number of patients in the two patients' subgroups (*early-stage* and *mid-advanced stage*) (38). Moreover, since all *early-stage* patients were also *drug-naïve*, we excluded possible confounding on voice recordings from chronic treatment with L-Dopa thus allowing the objective and automatic recognition of PD-related voice disorders *per se*. Concerning the specific speech tasks, we compared the sustained emission of a vowel and a sentence by using standardized procedures (11, 17–19, 22, 43) thus also verifying the effect of PD on voice samples of different complexity.

The clinical observation that 84% of the PD patients (68**%** of *early-stage* and 100% of *mid-advanced-stage* patients) manifested voice impairment (UPDRS-III-v ≥1), agrees with the estimated prevalence of hypokinetic dysarthria in PD, which ranges from 70 to 90% (1–4, 44). Furthermore, the severity of voice impairment correlated with disease duration and the overall motor disability and severity, and finally, with the degree of cognitive impairment in PD. Hence, our findings demonstrate that PD patients manifest voice disorders in the *early-stage* of the disease (2, 5), with significant worsening of speech over the course of the disease (1, 2).

The application of machine learning analysis showed that voice is abnormal in PD as demonstrated by high diagnostic accuracy in the discrimination of voices between PD patients and HS. Our findings confirm and expand preliminary machine learning studies only focused on specific methodological aspects of voice analysis, achieved in pre-existing datasets or in rather heterogeneus cohorts of patients with PD (24–26). Our study is therefore the first one to provide a thorought classification of voice in PD patients, according to the stage (i.e., *de novo*) and severity of the disease as well as the effect of chronic L-Dopa treatment. Also, supporting the biological plausibility of our results, the most relevant voice features selected by our machine learning algorithms (among the large dataset of features examined), include those previously identified by spectral analysis such as the fundamental frequency (3, 12–16, 26, 45). Moreover, our study showed for the first time significant clinico-instrumental correlations: the higher LR values attributed by machine learning, the longer the disease duration, the higher severity of motor symptoms, and finally the greater voice impairment in patients with PD. Hence, we demonstrated for the first time that the degree of voice changes in PD correlates with disease duration and severity and finally, LR values can be considered reliable scores to express the complexity of voice impairment in PD.

A further relevant finding of the study concerns the subclinical impairment of voice in *early-stage* PD as demonstrated by high statistical accuracy achieved by machine learning in discriminating *early-stage* patients from HS (2). Given that 32% of *early-stage* patients did not manifest a clinically overt voice impairment, we speculate that the high accuracy in discriminating *early-stage* patients and HS would reflect the ability of machine learning to recognize subclinical voice impairment in PD.

As the disease progresses, voice increasingly degrades in PD as demonstrated by our ROC analysis achieving high statistical accuracy in discriminating *mid-advanced-stage* patients OFF therapy from HS. Again, for the first time we demonstrate significant clinico-instrumental correlations: the higher LR values, the greater severity of voice symptoms in *mid-advanced-stage* patients.

Another important finding in this study concerns the effect of L-Dopa on voice abnormalities in PD which is still a matter of debate given previous reports on beneficial (28, 29, 31–33) or null effect (27, 30). We here demonstrated that L-Dopa exerts significant improvement of voice in *mid-advanced-stage* patients. Furthermore, our clinical evaluation allowed us to demonstrate that L-Dopa improved voice less than other motor symptoms, a finding pointing to the weaker clinical effect of L-Dopa on axial signs in PD, as also shown by the correlations between LEDDs and VHI as well as UPDRS-III-**v** (1, 27, 30). By using an objective and automatic voice analysis, we demonstrated the significant effect of L-Dopa on voice in PD as suggested by high diagnostic accuracy in the comparison of patients OFF and ON therapy. Still, we found for the first time significant clinico-instrumental correlations also in patients ON therapy: the greater LR values, the higher severity of motor symptoms. However, although L-Dopa improved voice in PD, it failed to restore it as demonstrated by high diagnostic accuracy in the discrimination between HS and patients ON therapy.

The diagnosis of PD is currently based on clinical examination with the aid of several standardized clinical scales (34). Hence, the development of innovative disease biomarkers in PD would gain tremendous advances in the field. According to the FDA, an ideal disease biomarker would imply the identification of a certain biological variable specific for PD and able to allow early and objective diagnosis and track the severity of the disease. Also, an ideal disease biomarker in PD would require a safe, easy, and cheap methodology enabling an accurate diagnosis of PD. A relevant finding here is that our machine learning algorithm can recognize PD even in the *early-stage* of the disease, track the disease severity and evaluate the symptomatic effect of L-Dopa using a safe, easy, and cheap methodology. Accordingly, the data reported in the present study would suggest the possible use of machine learning voice analysis as an innovative biomarker in PD.

A final comment deserves the specific speech tasks here used to assess voice in PD. In agreement with our previous studies (19, 22), when comparing voice samples during the emission of a vowel and a standardized sentence, our analysis disclosed similar ROC curves in PD. We therefore demonstrated a similar degree of PD-related voice impairment regardless of the complexity of the speech tasks used. Accordingly, given that the sustained emission of the vowel represents a language- and culture-free speech task, we suggest the voluntary emission of a vowel as the preferred speech task for the worldwide assessment of PD (19, 22).

We recognize that the present study has several limitations. As we have not recorded vocal samples in each patient serially, we cannot exclude the possibility of daily fluctuations in vocal features in PD. Also, in this study *early-stage* patients were slightly younger than *mid-advanced-stage* patients and HS. Hence, we cannot exclude that age differences between *early-stage* and *mid-advanced-stage* patients or HS would have contributed at least in part to the high accuracy achieved in the discrimination between the two subgroups of patients (19). Concerning the clinical-instrumental correlations, given that machine learning analysis requires a large amount of data, we speculate that future studies with larger sample size will report higher r values than those here reported. Furthermore, the uncertain association between specific aspects of *hypokinetic dysarthria* in PD (i.e., hypophonia, mono-pitch and mono-loudness speech) and the specific voice features selected by the machine learning algorithm requires further investigation in depth.

In conclusion, in the present study in a large and clinically well-characterized cohort of patients, we provide clinical and instrumental evidence supporting voice changes occurring early in PD and worsening significantly over the course of the disease. Also, L-Dopa improves but does not restore voice in PD. Overall, given that machine learning objectively recognizes PD even in the *early-stage* of the disease, tracks the disease severity and detects the effect of L-Dopa with previously unreported high diagnostic accuracy, we speculate that machine learning-based voice analysis would represent in a near future an innovative disease biomarker able to support the clinical management of PD. Lastly, we speculate that our study would promote the future homebound application of machine learning voice analysis for telemedicine approaches in PD.

## Data Availability Statement

All clinical and instrumental data are stored offline and are available on reasonable request to the corresponding author.

## Ethics Statement

The studies involving human participants were reviewed and approved by IRB of Tor Vergata University of Rome, Italy. The patients/participants provided their written informed consent to participate in this study.

## Author Contributions

AS, GC, FA, and GS: research project—conception and organization. FA, PDL, MSA-W, GDL, and SS: research project—execution. AS, GC, FA, and PL: statistical analysis—design. AS, FA, and PL: statistical analysis—execution. GC, AP, and GS: statistical analysis—review and critique. AS, GC, and FA: manuscript preparation—writing of the first draft. AP and GS: manuscript preparation—review and critique. All authors contributed to the article and approved the submitted version.

## Conflict of Interest

GC, GS, and AP are advisory members of VoiceWise S.r.l., spin-off company of University of Rome Tor Vergata (Rome, Italy) developing voice analysis solutions for diagnostic purposes. The remaining authors declare that the research was conducted in the absence of any commercial or financial relationships that could be construed as a potential conflict of interest.

## Publisher's Note

All claims expressed in this article are solely those of the authors and do not necessarily represent those of their affiliated organizations, or those of the publisher, the editors and the reviewers. Any product that may be evaluated in this article, or claim that may be made by its manufacturer, is not guaranteed or endorsed by the publisher.

## References

[1] Fabbri M Guimarães I Cardoso R Coelho M Guedes LC Rosa MM. Speech and voice response to a levodopa challenge in late-stage Parkinson's disease. Front Neurol. (2017) 8:432. 10.3389/fneur.2017.00432 28878734 PMC5572389

[2] Ma A Lau KK Thyagarajan D. Voice changes in Parkinson's disease: what are they telling us? J Clin Neurosci. (2020) 72:1–7. 10.1016/j.jocn.2019.12.029 31952969

[3] Rusz J Cmejla R Ruzickova H Ruzicka E. Quantitative acoustic measurements for characterization of speech and voice disorders in early untreated Parkinson's disease. J Acoust Soc Am. (2011) 129:350–67. 10.1121/1.3514381 21303016

[4] Ramig L Halpern A Spielman J Fox C Freeman K. Speech treatment in Parkinson's disease: randomized controlled trial (RCT): speech treatment in Parkinson's disease: RCT. Mov Disord. (2018) 33:1777–91. 10.1002/mds.27460 30264896 PMC6261685

[5] Fereshtehnejad S-M Yao C Pelletier A Montplaisir JY Gagnon J-F Postuma RB. Evolution of prodromal Parkinson's disease and dementia with Lewy bodies: a prospective study. Brain. (2019) 142:2051–67. 10.1093/brain/awz111 31111143

[6] Rusz J Hlavnička J Novotný M Tykalová T Pelletier A Montplaisir J. Speech biomarkers in rapid eye movement sleep behavior disorder and Parkinson disease. Ann Neurol. (2021) 90:62–75. 10.1002/ana.26085 33856074 PMC8252762

[7] Hlavnička J Cmejla R Tykalová T Šonka K RuŽička E Rusz J. Automated analysis of connected speech reveals early biomarkers of Parkinson's disease in patients with rapid eye movement sleep behaviour disorder. Sci Rep. (2017) 7:12. 10.1038/s41598-017-00047-5 28144037 PMC5428345

[8] Rusz J Tykalová T Novotný M Zogala D RuŽička E Dušek P. Automated speech analysis in early untreated Parkinson's disease: Relation to gender and dopaminergic transporter imaging. Eur J Neurol. (2021) 29:81–90. 10.1111/ene.15099 34498329

[9] Arora S Baig F Lo C Barber TR Lawton MA Zhan A. Smartphone motor testing to distinguish idiopathic REM sleep behavior disorder, controls, and PD. Neurology. (2018) 91:e1528–38. 10.1212/WNL.0000000000006366 30232246 PMC6202945

[10] Antonini A Abbruzzese G Ferini-Strambi L Tilley B Huang J Stebbins GT. Validation of the Italian version of the Movement Disorder Society–Unified Parkinson's Disease Rating Scale. Neurol Sci. (2013) 34:683–7. 10.1007/s10072-012-1112-z 22678179

[11] Rusz J Tykalova T Ramig LO Tripoliti E. Guidelines for speech recording and acoustic analyses in dysarthrias of movement disorders. Mov Disord. (2020) 36:803–14. 10.1002/mds.28465 33373483

[12] Bhuta T Patrick L Garnett JD. Perceptual evaluation of voice quality and its correlation with acoustic measurements. J Voice. (2004) 18:299–304. 10.1016/j.jvoice.2003.12.004 15331102

[13] Gamboa J Jiménez-Jiménez FJ Nieto A Montojo J Ortí-Pareja M Molina JA. Acoustic voice analysis in patients with Parkinson's disease treated with dopaminergic drugs. J Voice. (1997) 11:314–20. 10.1016/S0892-1997(97)80010-0 9297676

[14] Rusz J Tykalová T Klempír J Cmejla R RuŽička E. Effects of dopaminergic replacement therapy on motor speech disorders in Parkinson's disease: longitudinal follow-up study on previously untreated patients. J Neural Transm. (2016) 123:379–87. 10.1007/s00702-016-1515-8 26843071

[15] Rusz J Cmejla R RuŽičková H Klempír J Majerová V Picmausová J. Evaluation of speech impairment in early stages of Parkinson's disease: a prospective study with the role of pharmacotherapy. J Neural Transm. (2013) 120:319–29. 10.1007/s00702-012-0853-4 22772465

[16] Tanaka Y Nishio M Niimi S. Vocal acoustic characteristics of patients with Parkinson's disease. Folia Phoniatr Logop. (2011) 63:223–30. 10.1159/000322059 21212679

[17] Asci F Costantini G Saggio G Suppa A. Fostering voice objective analysis in patients with movement disorders. Mov Disord. (2021) 36:1041. 10.1002/mds.28537 33851751

[18] Asci F Costantini G Di Leo P Saggio G Suppa A. Reply to: Reproducibility of voice analysis with machine learning. Mov Disord. (2021) 36:1283–4. 10.1002/mds.28601 33991448

[19] Asci F Costantini G Di Leo P Zampogna A Ruoppolo G Berardelli A. Machine-learning analysis of voice samples recorded through smartphones: the combined effect of ageing and gender. Sensors. (2020) 20:5022. 10.3390/s20185022 32899755 PMC7570582

[20] Hegde S Shetty S Rai S Dodderi T. A survey on machine learning approaches for automatic detection of voice disorders. J Voice. (2019) 33:947.e11–947.e33. 10.1016/j.jvoice.2018.07.014 30316551

[21] Suppa A Asci F Saggio G Di Leo P Zarezadeh Z Ferrazzano G. Voice analysis with machine learning: one step closer to an objective diagnosis of essential tremor. Mov Disord. (2021) 36:1401–10. 10.1002/mds.28508 33528037

[22] Suppa A Asci F Saggio G Marsili L Casali D Zarezadeh Z. Voice analysis in adductor spasmodic dysphonia: objective diagnosis and response to botulinum toxin. Parkinsonism Relat Disord. (2020) 73:23–30. 10.1016/j.parkreldis.2020.03.012 32222482

[23] Vu M-AT Adali T Ba D Buzsáki G Carlson D Heller K. A shared vision for machine learning in neuroscience. J Neurosci. (2018) 38:1601–7. 10.1523/JNEUROSCI.0508-17.2018 29374138 PMC5815449

[24] Karapinar Senturk Z. Early diagnosis of Parkinson's disease using machine learning algorithms. Med Hypoth. (2020) 138:109603. 10.1016/j.mehy.2020.109603 32028195

[25] Sakar CO Kursun O. Telediagnosis of Parkinson's disease using measurements of dysphonia. J Med Syst. (2010) 34:591–9. 10.1007/s10916-009-9272-y 20703913

[26] Vaiciukynas E Verikas A Gelzinis A Bacauskiene M. Detecting Parkinson's disease from sustained phonation and speech signals. PLoS ONE. (2017) 12:e0185613. 10.1371/journal.pone.0185613 28982171 PMC5628839

[27] Cavallieri F Budriesi C Gessani A Contardi S Fioravanti V Menozzi E. Dopaminergic treatment effects on dysarthric speech: acoustic analysis in a cohort of patients with advanced Parkinson's disease. Front Neurol. (2020) 11:616062. 10.3389/fneur.2020.616062 33613419 PMC7892955

[28] Lechien JR Delsaut B Abderrakib A Huet K Delvaux V Piccaluga M. Orofacial strength and voice quality as outcome of levodopa challenge test in Parkinson disease. Laryngoscope. (2020) 130:E896–903. 10.1002/lary.28645 32239775

[29] Norel R Agurto C Heisig S Rice JJ Zhang H Ostrand R. Speech-based characterization of dopamine replacement therapy in people with Parkinson's disease. NPJ Parkinsons Dis. (2020) 6:12. 10.1038/s41531-020-0113-5 32566741 PMC7293295

[30] Pinho P Monteiro L Soares MFdP Tourinho L Melo A Nóbrega AC. Impact of levodopa treatment in the voice pattern of Parkinson's disease patients: a systematic review and meta-analysis. CoDAS. (2018) 30:e20170200. 10.1590/2317-1782/20182017200 30304100

[31] Sanabria J Ruiz PG Gutierrez R Marquez F Escobar P Gentil M. The effect of levodopa on vocal function in Parkinson's disease. Clin Neuropharmacol. (2001) 24:99–102. 10.1097/00002826-200103000-00006 11307045

[32] Wolfe VI Garvin JS Bacon M Waldrop W. Speech changes in Parkinson's disease during treatment with L-DOPA. J Commun Disord. (1975) 8:271–9. 10.1016/0021-9924(75)90019-2 802977

[33] Rusz J Tykalova T Novotny M Zogala D Sonka K Ruzicka E. Defining speech subtypes in de novo parkinson disease: response to long-term levodopa therapy. Neurology. (2021) 97:e2124–35. 10.1212/WNL.0000000000012878 34607922

[34] Postuma RB Berg D Stern M Poewe W Olanow CW Oertel W. MDS clinical diagnostic criteria for Parkinson's disease. Mov Disord. (2015) 30:1591–601. 10.1002/mds.26424 26474316

[35] Folstein MF Folstein SE McHugh PR. “Mini-mental state”. A practical method for grading the cognitive state of patients for the clinician. J Psychiatr Res. (1975) 12:189–98. 10.1016/0022-3956(75)90026-6 1202204

[36] Hamilton M. A rating scale for depression. J Neurol Neurosurg Psychiatry. (1960) 23:56–62. 10.1136/jnnp.23.1.56 14399272 PMC495331

[37] Schindler A Ottaviani F Mozzanica F Bachmann C Favero E Schettino I. Cross-cultural adaptation and validation of the voice handicap index into Italian. J Voice. (2010) 24:708–14. 10.1016/j.jvoice.2009.05.006 20083383

[38] Hacker ML Turchan M Heusinkveld LE Currie AD Millan SH Molinari AL al. Deep brain stimulation in early-stage Parkinson disease: five-year outcomes. Neurology. (2020) 95:e393–401. 10.1212/WNL.0000000000009946 32601120 PMC7455319

[39] Eyben F Wöllmer M Schuller B. Opensmile: the munich versatile and fast open-source audio feature extractor. In: Proceedings of the International Conference on Multimedia - MM '10. Firenze: ACM Press (2010). p. 1459 10.1145/1873951.1874246

[40] Hall M. Correlation-based feature selection for machine learning. Dep Comput Sci. (2000) 19:1–198.

[41] Kullback S Leibler RA. On Information and sufficiency. Ann Math Statist. (1951) 22:79–86. 10.1214/aoms/1177729694

[42] Saggio G Costantini G. Worldwide healthy adult voice baseline parameters: A comprehensive review. J Voice. (2020) S0892-1997(20)30328-3. 10.1016/j.jvoice.2020.08.028. [Epub ahead of print]. 33039203

[43] Tripoliti E. Voice tremor and acoustic analysis: finding harmony through the waves. Clin Neurophysiol. (2020) 131:1144–5. 10.1016/j.clinph.2020.02.017 32199728

[44] Harel B Cannizzaro M Snyder PJ. Variability in fundamental frequency during speech in prodromal and incipient Parkinson's disease: a longitudinal case study. Brain Cogn. (2004) 56:24–9. 10.1016/j.bandc.2004.05.002 15380872

[45] Rahman A Rizvi SS Khan A Afzaal Abbasi A Khan SU Chung T-S. Parkinson's disease diagnosis in cepstral domain using MFCC and dimensionality reduction with svm classifier. Mobile Inform Syst. (2021) 2021:e8822069. 10.1155/2021/88220692021

